# Supplementary material for: A sex-stratified analysis of the genetic architecture of human brain anatomy
Source: Nat Commun. 2024 Sep 13;15:8041. doi: 10.1038/s41467-024-52244-2 (PMC11399304; doi:10.1038/s41467-024-52244-2)
Supplement: Supplementary file 1 — Supplementary Information [file 41467_2024_52244_MOESM1_ESM.pdf]

Supplementary Information

A sex-stratified analysis of the genetic architecture of human brain anatomy

Rebecca Shafee[1], Dustin Moraczewski[2], Siyuan Liu[1], Travis Mallard[3,4], Adam Thomas[2], Armin Raznahan*[1]*

1. *Section on Developmental Neurogenomics, Human Genetics Branch, NIMH Intramural Research Program, NIH, Bethesda, MD, 20892*
2. *Data Science and Sharing Team, NIMH, NIH, Bethesda, MD 20892*
3. *Psychiatric and Neurodevelopmental Genetics Unit, Center for Genomic Medicine, Massachusetts General Hospital, Boston, MA 02114*
4. *Department of Psychiatry, Harvard Medical School, Boston 02215*

*Corresponding authors

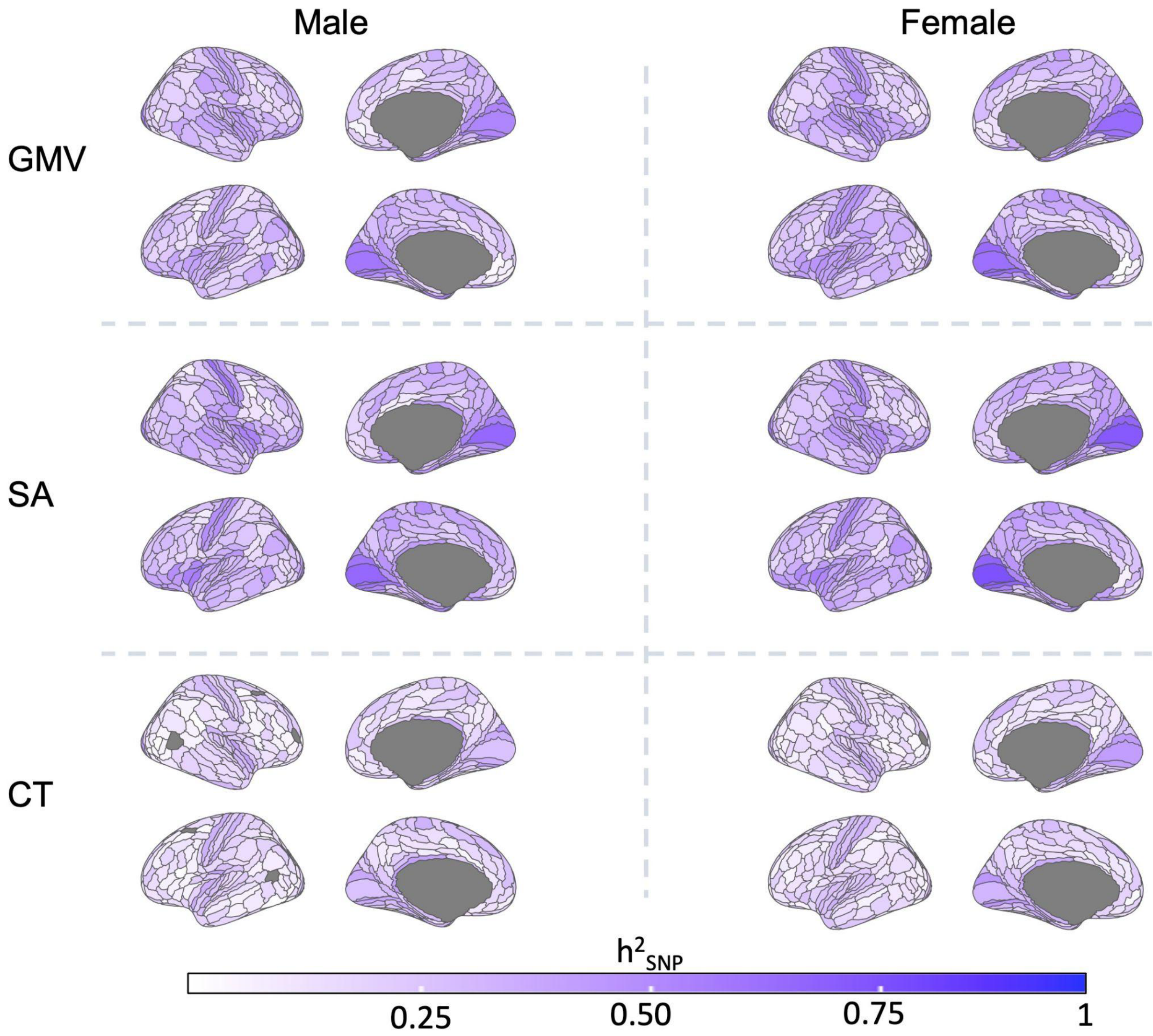

Supplementary Figure 1: **SNP-based heritability (h^2_{SNP}) of gray matter volume (GMV), surface area (SA) and cortical thickness (CT) in the UKB**. Similar to **Figure 2** in the main text but shows results for both hemispheres. Sex-specific spatial maps of SNP-based total heritability (h^2_{SNP}) of regional cortical gray matter volume (GMV), surface area (SA) and cortical thickness (CT) as measured by GCTA (Supplementary Data 2,3,4). Sample sizes for each category of phenotypes are provided in Supplementary Data 1. Source data are provided as Source Data file.

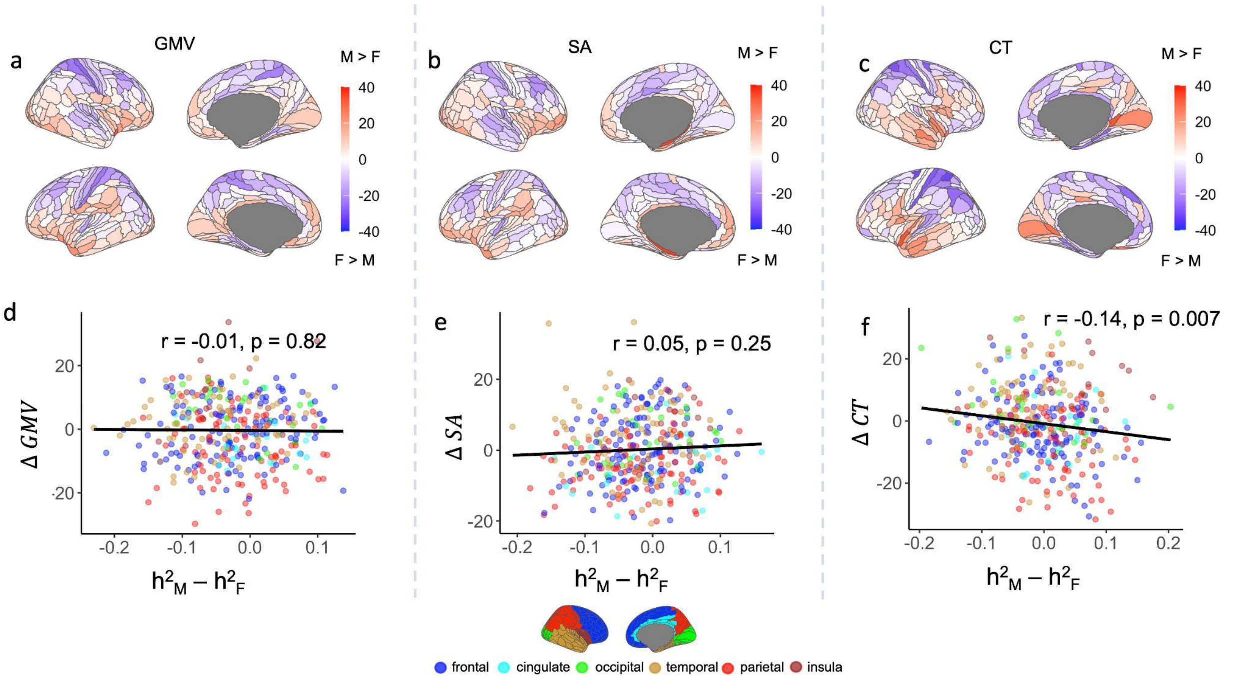

Supplementary Figure 2: **Comparison between phenotypic sex difference and sex difference in h^2_{SNP}.** (**a, b, c)** Phenotypic sex differences for gray matter volume- GMV (**a**), surface area - SA (**b**) and cortical thickness - CT (**c**) shown as t-statistics of the coefficient of the "sex" term in a linear model which also controlled for age, age^2, scanner position, Euler number, scan center and corresponding global phenotypes (Methods, in main text). Red indicates higher value in male participants whereas blue indicates higher values in female participants. Sample sizes for each category of phenotypes are provided in Supplementary Data 1. (**d**, **e**, **f)** scatter plots of sex-difference in h^2_{SNP} (male - female) vs. phenotypic sex-difference for GMV (**d**), SA (**e**) and CT (**f**). In these plots a positive coordinate indicates higher value in the male group. The points are colored by cortical lobe assignment of the HCP parcellation as shown in the panel below the scatter plots. Pearson's correlation coefficients and corresponding p-values are also reported. Source data are provided as Source Data file.

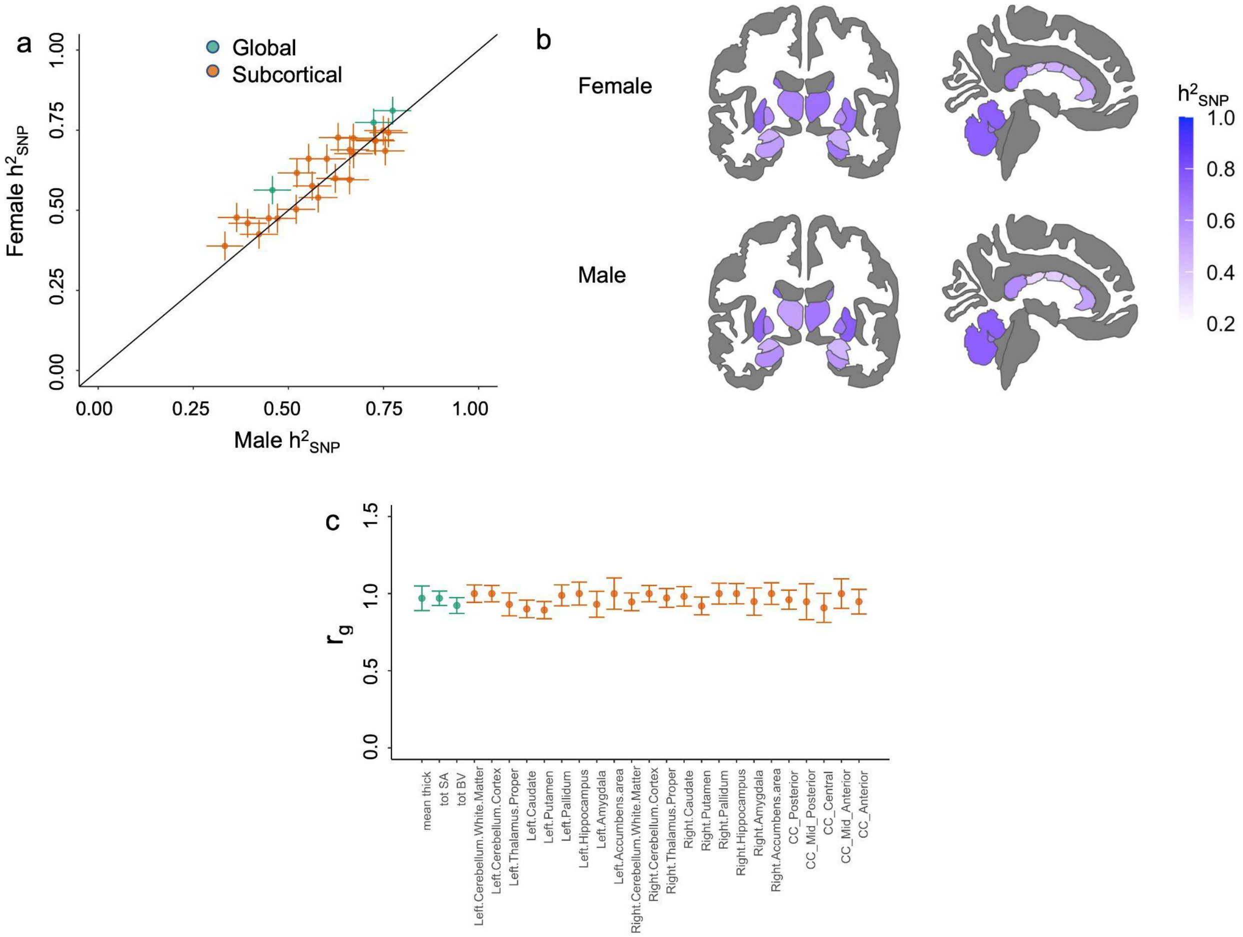

Supplementary Figure 3: Heritability (h^2_{SNP}) and genetic correlation (r_g) in subcortical and global phenotypes. **a**) Scatter plot showing male and female h^2_{SNP} for 3 global and 23 subcortical volumes. Data points show estimated h^2_{SNP} +/- SE (Source Data) **b**) h^2_{SNP} of subcortical structure volumes. **c)** Autosomal genetic correlation (r_g) between male and female participants for the same phenotypes (as in **a**) as calculated by GCTA. Data points show rg +/- SE of rg as calculated by GCTA. For subcortical volumes: N = 14071 male participants and N = 15831 female participants; for global measures: N = 14484 male participants and N = 1623 female participants. Source data are provided as Source Data file.

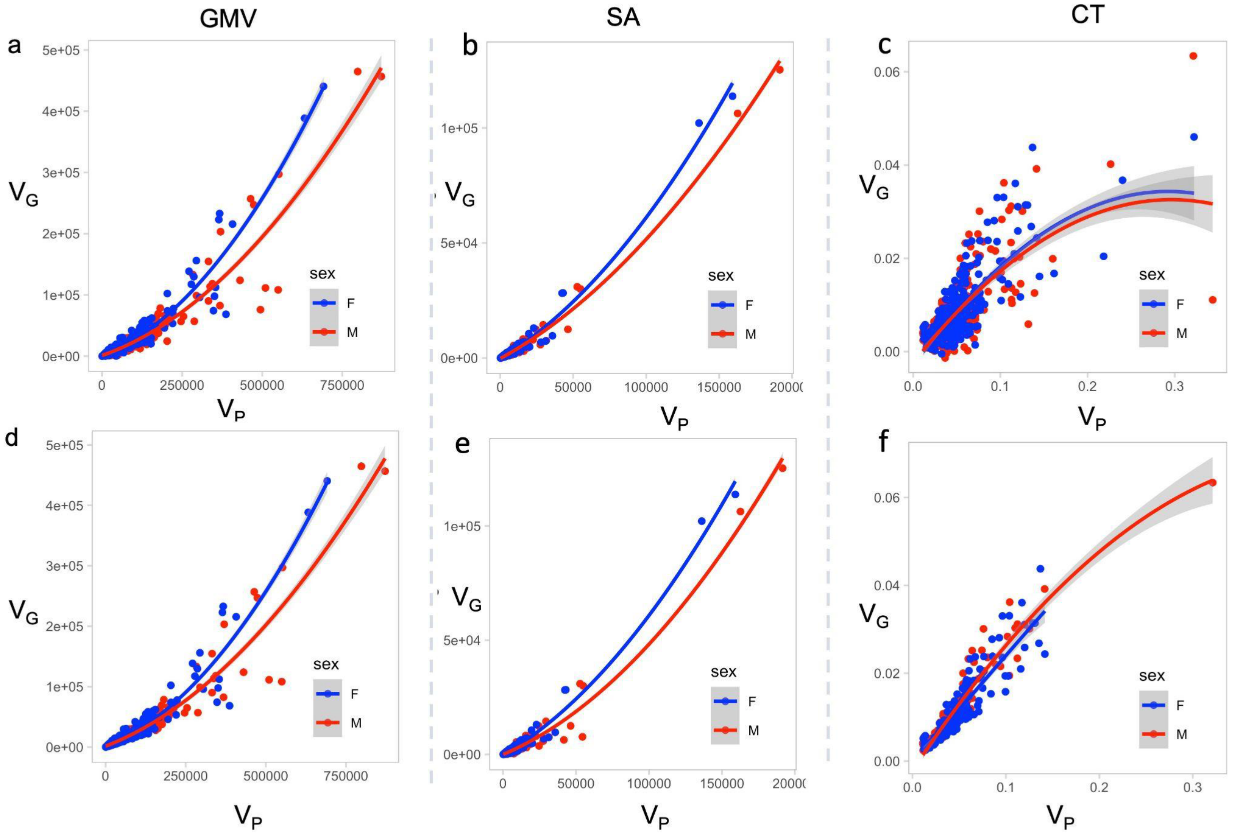

Supplementary Figure 4: **Scatter plots comparing genetic variance V_G and phenotypic variance V_p in both sexes**. (**a**, **b**, **c)** show these values for each region in the HCP parcellation (180 regions in each hemisphere) for gray matter volume (GMV), surface area (SA) and cortical thickness (CT) corrected for age, age^2, scanner position, Euler number, scan center and corresponding global phenotypes. (**d**, **e**, **f)** show similar plots but for regions which showed multiple-testing corrected (MTC) significant h^2_{SNP} ($p < p_{MTC} = 1.4e-4$) only. The blue and red solid lines show sex-specific fits to the data for which the coefficients can be found in Supplementary Data 8. Source data are provided as Source Data file.

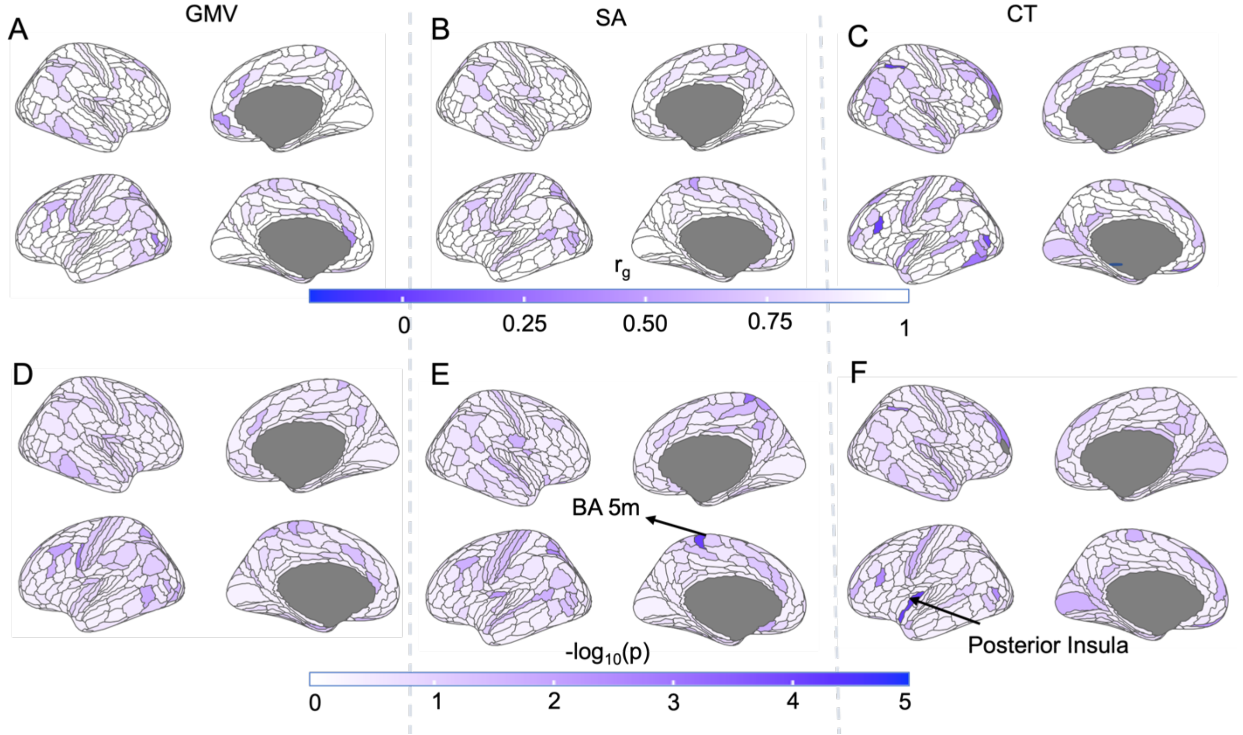

Supplementary Figure 5: **Between-sex genetic correlations (r_g) in brain anatomy.** Same as Figure 3 in main text, but also including regions with h^2_{SNP} p-value >0.05. Genetic correlation r_g for gray. Matter volume - GMV(**a**), surface area - SA(b) and cortical thickness - CT(c) and p-value of r_g <1 (calculated from a log likelihood test in GCTA) for GMV, SA and CT (d, e, f) are shown. Only two regions showed significant r_g < 1 after multiple-testing correction (MTC) ($p < p_{MTC}$ = 0.05/360 = 1.4e-4): one in SA (superior parietal lobule medial Brodmann area 5, 5m, p= as 7.9 x $10^{-5,}$ r_g = 0.50 +/- 0.11) and one in CT (posterior insula, PoI1, p = 7.7e-5, r_g = 0.46 +/- 0.11). All regional phenotypes were corrected for corresponding global measures (mean thickness, total surface area and total brain volume) prior to calculating r_g. Source data are provided as Source Data file.

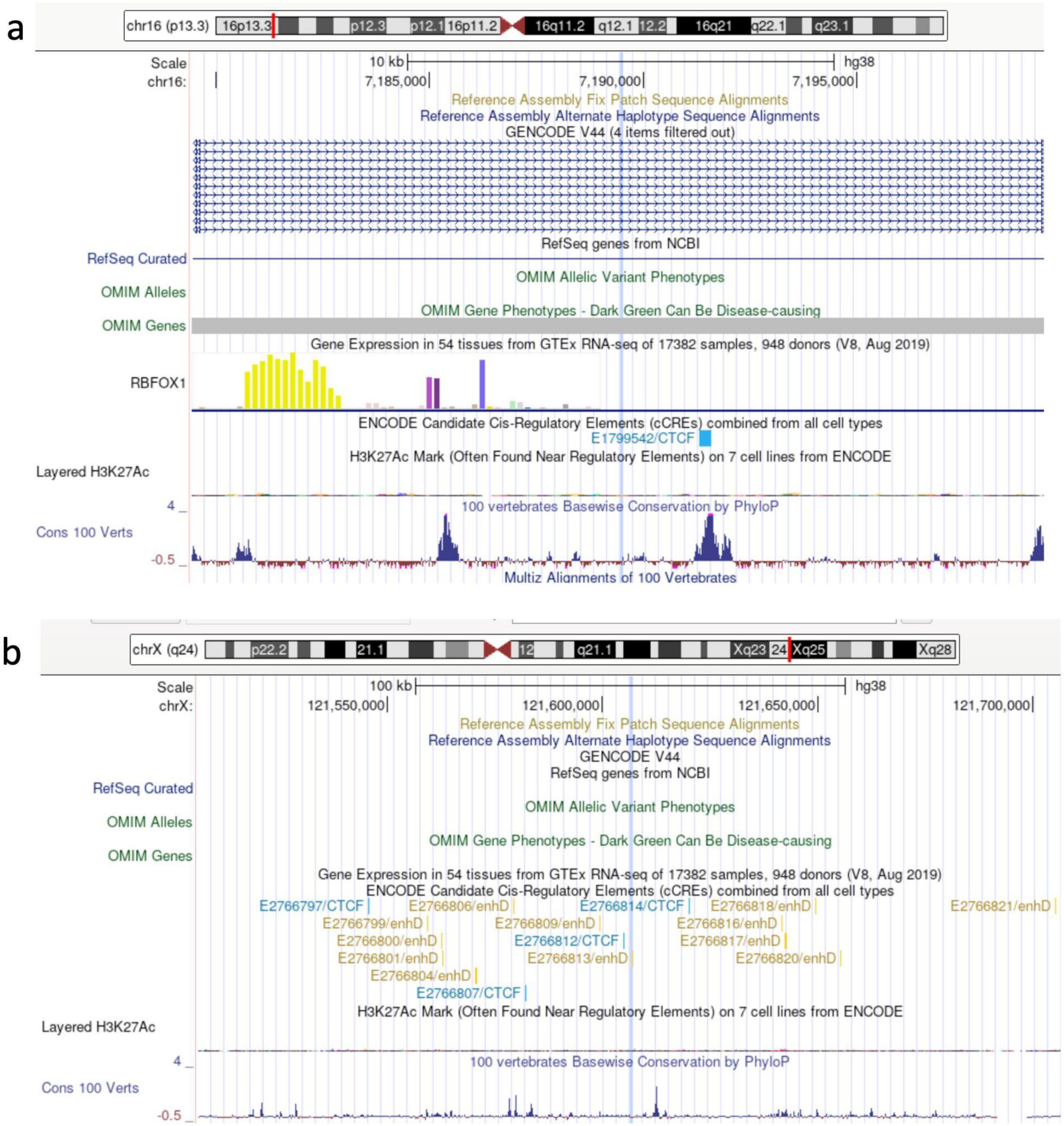

Supplementary Figure 6: **Plots showing the genomic region corresponding to the two SNPs showing sex-differentiated effects in the brain phenotypes**, **a)** chr16:rs113078989 and **b)** chrX:rs747862348. Figures were generated using https://genome.ucsc.edu/. Chr16:rs113078989 is an intronic SNP of *RBFOX1* but chrX:rs747862348 cannot be mapped to any gene within 100 kb window using positional mapping.
