# Supplementary material for: A sex-stratified analysis of the genetic architecture of human brain anatomy
Source: Nat Commun. 2024 Sep 13;15:8041. doi: 10.1038/s41467-024-52244-2 (PMC11399304; doi:10.1038/s41467-024-52244-2)
Supplement: Supplementary file 3 — Description Of Additional Supplementary Files [file 41467_2024_52244_MOESM3_ESM.pdf]

Description of Additional Supplementary Files

File Name: Supplementary Data 1
Description: Demographic and phenotypes information for the study.

File Name: Supplementary Data 2
Description: SNP heritability of regional cortical gray matter volume (GMV)

File Name: Supplementary Data 3
Description: SNP heritability of regional cortical gray matter surface area (SA)

File Name: Supplementary Data 4
Description: SNP heritability of regional cortical thickness (CT)

File Name: Supplementary Data 5
Description: SNP heritability of global phenotypes

File Name: Supplementary Data 6
Description: SNP heritability of subcortical volumes

File Name: Supplementary Data 7
Description: Sex-difference in genetic variance VG, phenotypic variance VP and environmental variance VE

File Name: Supplementary Data 8
Description: Models investigating sex-difference in the relationship between genetic variance VG and phenotypic variance VP

File Name: Supplementary Data 9
Description: Between-sex genetic correlation (rg) of regional gray matter volume (GMV)

File Name: Supplementary Data 10
Description: Between-sex genetic correlation (rg) of regional surface area (SA)

File Name: Supplementary Data 11
Description: Between-sex genetic correlation (rg) of regional cortical thickness (CT)

File Name: Supplementary Data 12
Description: Between-sex genetic correlation (rg) of global phenotypes

File Name: Supplementary Data 13
Description: Between-sex genetic correlation (rg) of subcortical volumes

File Name: Supplementary Data 14
Description: SNPs passing the "strict" significance threshold in SNP-level sex difference analyses

File Name: Supplementary Data 15
Description: Number of SNPs passing the "relaxed" significance threshold in SNP-level sex difference analyses

File Name: Supplementary Data 16
Description: Candidate SNPs and mapped genes from FUMA analysis of SNPs passing the "relaxed" threshold for regional GMV

File Name: Supplementary Data 17
Description: Candidate SNPs and mapped genes from FUMA analysis of SNPs passing the "relaxed" threshold for regional SA

File Name: Supplementary Data 18
Description: Candidate SNPs and mapped genes from FUMA analysis of SNPs passing the "relaxed" threshold for regional CT

File Name: Supplementary Data 19
Description: Candidate SNPs and mapped genes from FUMA analysis of SNPs passing the "relaxed" threshold for subcortical volumes

File Name: Supplementary Data 20
Description: Candidate SNPs and mapped genes from FUMA analysis of all SNPs passing the "relaxed" threshold for regional GMV

File Name: Supplementary Data 21
Description: Overlap between RBFOX1 sex-biased regions and functional networks

File Name: Supplementary Data 22
Description: Accession codes for the GWAS summary statistics deposited to public repository
