# Supplementary material for: A sex-stratified analysis of the genetic architecture of human brain anatomy
Source: Nat Commun. 2024 Sep 13;15:8041. doi: 10.1038/s41467-024-52244-2 (PMC11399304; doi:10.1038/s41467-024-52244-2)
Supplement: Supplementary file 26 — Reporting Summary [file 41467_2024_52244_MOESM26_ESM.pdf]

nature portfolio

Corresponding author(s): Dr. Armin Raznahan, Dr. Rebecca Shafee

Last updated by author(s): Jun 21, 2024

Reporting Summary

Nature Portfolio wishes to improve the reproducibility of the work that we publish. This form provides structure for consistency and transparency in reporting. For further information on Nature Portfolio policies, see our Editorial Policies and the Editorial Policy Checklist.

Statistics

For all statistical analyses, confirm that the following items are present in the figure legend, table legend, main text, or Methods section.

n/a	Confirmed	
☐	☒	The exact sample size (*n*) for each experimental group/condition, given as a discrete number and unit of measurement
☒	☐	A statement on whether measurements were taken from distinct samples or whether the same sample was measured repeatedly
☐	☒	The statistical test(s) used AND whether they are one- or two-sided<br>*Only common tests should be described solely by name; describe more complex techniques in the Methods section.*
☐	☒	A description of all covariates tested
☐	☒	A description of any assumptions or corrections, such as tests of normality and adjustment for multiple comparisons
☐	☒	A full description of the statistical parameters including central tendency (e.g. means) or other basic estimates (e.g. regression coefficient) AND variation (e.g. standard deviation) or associated estimates of uncertainty (e.g. confidence intervals)
☐	☒	For null hypothesis testing, the test statistic (e.g. *F*, *t*, *r*) with confidence intervals, effect sizes, degrees of freedom and *P* value noted<br>*Give P values as exact values whenever suitable.*
☒	☐	For Bayesian analysis, information on the choice of priors and Markov chain Monte Carlo settings
☒	☐	For hierarchical and complex designs, identification of the appropriate level for tests and full reporting of outcomes
☐	☒	Estimates of effect sizes (e.g. Cohen's *d*, Pearson's *r*), indicating how they were calculated

Our web collection on statistics for biologists contains articles on many of the points above.

Software and code

Policy information about availability of computer code

Data collection	No software was used for data collection.
Data analysis	R v.3.6.0, Plink v.1.9 and v2.0, GCTA vl.93.0, FreeSurfer 6.0.1, FUMA (https://fuma.ctglab.nl/) version 1.5.2, 1.5.3, 1.5.4 ; R packages: ggseg (https://github.com/ggseg/ggseg) and ggsegGlasser (https://github.com/ggseg/ggsegGlasser/), myManhattan (https://github.com/alfonsosaera/myManhattan/), qqman (https://github.com/stephenturner/qqman)

For manuscripts utilizing custom algorithms or software that are central to the research but not yet described in published literature, software must be made available to editors and reviewers. We strongly encourage code deposition in a community repository (e.g. GitHub). See the Nature Portfolio guidelines for submitting code & software for further information.

Data

Policy information about availability of data

All manuscripts must include a data availability statement. This statement should provide the following information, where applicable:

- Accession codes, unique identifiers, or web links for publicly available datasets
- A description of any restrictions on data availability
- For clinical datasets or third party data, please ensure that the statement adheres to our policy

The sex-stratified genome-wide association data generated in this study have been deposited in the https://www.ebi.ac.uk/gwas/ database under accession codes which may be found in Supplementary Data 22. The imputed genotype data and brain phenotypes in UK Biobank can be obtained by submitting an application at

https://www.ukbiobank.ac.uk/enable-your-research/apply-for-access. The SNP-heritability and genetic correlation data generated in this study are provided in the Supplementary Data and Source Data files.

Research involving human participants, their data, or biological material

Policy information about studies with human participants or human data. See also policy information about sex, gender (identity/presentation), and sexual orientation and race, ethnicity and racism.

Reporting on sex and gender	Our study investigates differences in the genetic architecture of brain structures for the two biological sex groups- XY (males) and XX (females). UK Biobank provided self-reported sex data for individuals which were combined with genotype-based sex information and samples with inconsistencies were excluded. We report results for each biological sex group (heritability, GWAS) and compare results between the sex-groups to investigate sex-difference. In our sample there were 14534 males and 16294 females.
Reporting on race, ethnicity, or other socially relevant groupings	We restricted the sample to participants of non-Hispanic European ancestry with processed neuroimaging data to the potential influence of population stratification in the genetic analyses.
Population characteristics	The UK Biobank (UKB) is a large-scale biomedical study designed to investigate the influence of genetic and environmental variables on human health and wellbeing. All participants provided informed consent, were genotyped, and a subset of participants also took part in a MRI protocol, which was the sample selected for our work. This sample is one of the largest individual-level datasets with both MRI and genotype data which are accessible by application and was ideal for our study of sex-difference in the genetic architecture of brain anatomy. The UK Biobank is largely representative of the UK population. The final sample size for the present study was 14534 males, 16294 females , mean age: males = 65.1 yrs, females = 63.7 yrs). Details of the sampling strategy for UK Biobank can be found at https://
Recruitment	The UK Biobank is a large population-based biobank that is largely representative of the UK population. Details on the sampling strategy can be found at https://www.ukbiobank.ac.uk, as well as in studies previously published by the original UK Biobank investigators.
Ethics oversight	This research was supported by the Intramural Research Program of the National Institute of Mental Health (ZIA MH002949-07, ZIC MH002960), and conducted using the UK Biobank Resource under Application Number 22875. Ethical approval for the UK Biobank study was obtained by the original investigators.

Note that full information on the approval of the study protocol must also be provided in the manuscript.

Field-specific reporting

Please select the one below that is the best fit for your research. If you are not sure, read the appropriate sections before making your selection.

- [x] Life sciences
- [ ] Behavioural & social sciences
- [ ] Ecological, evolutionary & environmental sciences

For a reference copy of the document with all sections, see nature.com/documents/nr-reporting-summary-flat.pdf

Life sciences study design

All studies must disclose on these points even when the disclosure is negative.

Sample size	Details of the sampling strategy for UK Biobank can be found at https://www.ukbiobank.ac.uk/media/gnkeyh2q/study-rationale.pdf, as well as in studies previously published by the original UK Biobank investigators. We selected the UK Biobank as our analytic sample, as it is (1) the largest sample with the types of data required for these analyses, and (2) well-powered for GREML-based heritability analyses and GWAS. This was an exploratory analysis and prior to the analysis we confirmed that the size was large enough for GREML-based heritability analysis (which requires N > 3000 for SE ~ 0.1) and genome-wide association analyses (past MRI studies in UKB had N > 8000).
Data exclusions	We made use of all available data that passed quality control thresholds. Data were only excluded if the data were considered low quality per standard conventions in the field. All data exclusion criteria are thoroughly described in the Methods section of the manuscript.
Replication	The sample size requirements of imaging genetics analyses are such that we currently lack suitable samples for fully independent replication of findings in the UK Biobank.
Randomization	No participants were involved in the current study
Blinding	No participants were involved in the current study.

Reporting for specific materials, systems and methods

We require information from authors about some types of materials, experimental systems and methods used in many studies. Here, indicate whether each material, system or method listed is relevant to your study. If you are not sure if a list item applies to your research, read the appropriate section before selecting a response.

Materials & experimental systems

n/a	Involved in the study
☒	☐ Antibodies
☒	☐ Eukaryotic cell lines
☒	☐ Palaeontology and archaeology
☒	☐ Animals and other organisms
☒	☐ Clinical data
☒	☐ Dual use research of concern
☒	☐ Plants

Methods

n/a	Involved in the study
☒	☐ ChIP-seq
☒	☐ Flow cytometry
☒	☐ MRI-based neuroimaging

Plants

Seed stocks

Report on the source of all seed stocks or other plant material used. If applicable, state the seed stock centre and catalogue number. If plant specimens were collected from the field, describe the collection location, date and sampling procedures.

Novel plant genotypes

Describe the methods by which all novel plant genotypes were produced. This includes those generated by transgenic approaches, gene editing, chemical/radiation-based mutagenesis and hybridization. For transgenic lines, describe the transformation method, the number of independent lines analyzed and the generation upon which experiments were performed. For gene-edited lines, describe the editor used, the endogenous sequence targeted for editing, the targeting guide RNA sequence (if applicable) and how the editor was applied.

Authentication

Describe any authentication procedures for each seed stock used or novel genotype generated. Describe any experiments used to assess the effect of a mutation and, where applicable, how potential secondary effects (e.g. second site T-DNA insertions, mosiacism, off-target gene editing) were examined.
